# Continuous Fermentation of Wheat Straw Hydrolysate by *Clostridium tyrobutyricum* with In-Situ Acids Removal

**DOI:** 10.1007/s12649-015-9348-5

**Published:** 2015-01-29

**Authors:** G. N. Baroi, I. V. Skiadas, P. Westermann, H. N. Gavala

**Affiliations:** Department of Chemistry and Bioscience, Section for Sustainable Biotechnology, Aalborg University (AAU), A C Meyers Vænge 15, 2450 Copenhagen SV, Denmark

**Keywords:** Butyric acid, *Clostridium tyrobutyricum*, Fermentation, Lignocellulosic biomass, Reverse electro enhanced dialysis, Wheat straw

## Abstract

The present study focused on fermentative butyric acid production by *Clostridium tyrobutyricum* from pre-treated and hydrolysed wheat straw (PHWS) based on continuous operation mode and in situ acids extraction by reverse electro enhanced dialysis (REED). Different dilutions of PHWS in a synthetic medium (60–100 % v/v) were tested. It was found that continuous fermentation of PHWS greatly enhanced the sugar consumption rates and butyric acid productivity compared to batch tests, while application of REED enhanced them even further. Specifically, applying combined continuous operation mode and REED system for the fermentation of 70 % PHWS resulted in 19- and 53-fold higher glucose (1.37 g L^−1^ h^−1^) and xylose (0.80 g L^−1^ h^−1^) consumption rates, respectively, compared to those obtained by batch processing. Fermentation of 100 % PHWS continued unhindered with just urea and K_2_HPO_4_ added with butyric acid production rate, yield and selectivity being 1.30 g L^−1^ h^−1^, 0.45 g g^−1^ sugars and 0.88 g g^−1^ acids, respectively. These results were also confirmed in a 20 L pilot plant bioreactor system.

## Introduction

A major step towards the development of a sustainable industrial society is a shift from petroleum-based resources to renewable resources. An ongoing effort is focused on developing bio-refineries as an alternative way of producing fuels and chemical building-blocks from renewable resources [1]. Thus, today’s organic residues and wastes may become tomorrow’s platforms for a variety of products for industrial use. Butyric acid fermentation has been discussed and investigated in the last decade due to the wide application of butyric acid in chemical, pharmaceutical and food industries [2, 3]. Compared to other microbial species, *Clostridium tyrobutyricum* is a strong candidate for biological production of butyric acid as it has a high selectivity and high tolerance to butyric acid [2, 3].

Studies focusing on continuous fermentation by *C. tyrobutyricum* are scarce in the international literature although *C. tyrobutyricum* is the most studied strain for butyric acid production. A reason for this could be that most industrial biotechnological processes so far are based on batch or fed-batch operation mode, despite the fact that continuous processing often results in higher productivities compared to batch/fed-batch processing. Villadsen [4] states that this is mainly due to the fact that the large-scale equipment is very much like it was in the 1940s while “companies themselves, being so happy with the order-of-magnitude increases in yield that are obtained by molecular biology that they overlook simpler methods for developing better production methods in large-scale”. This approach might still be cost-efficient for production of high-value chemicals and pharmaceuticals; however, for production of bulk chemicals of relatively low value, other more efficient methods could potentially bring us faster to a sustainable, bio-based economy.

Michel-Savin et al. [5, 6] have tested continuous butyric acid fermentation with *C. tyrobutyricum* grown on synthetic medium at a relatively low concentration of glucose (30–47 g L^−1^). In Michel-Savin et al. [5] a butyric acid productivity of 1.94 g L^−1^ h^−1^ was reported with a butyric acid selectivity and yield of 0.88 and 0.37 g g^−1^, respectively. These figures corresponded to a butyric acid concentration of 9.7 g L^−1^. When cell recycling was applied [6], the productivity increased to 9.5 g L^−1^ h^−1^ corresponding to a butyric acid concentration of 29.7 g L^−1^. Continuous butyric acid fermentation by *C. tyrobutyricum* on a synthetic glucose medium with partial cell recycling was also studied in Du et al. [7]. A productivity of 1.13 g L^−1^ h^−1^ was achieved, accompanied by 0.95 and 0.45 g g^−1^ butyric acid selectivity and yield, respectively. The concentration of butyric acid in the fermentor was 8 g L^−1^.

Productivity and yield of butyric acid is negatively affected by product inhibition at elevated concentrations [5]. Continuous in situ acid removal could be applied in order to overcome the inhibition caused by butyric acid accumulation. In this respect, in situ electrodialysis has been applied for lactic [8], acetic, propionic [9] and butyric acid extraction [10] and higher productivity was reported in all cases. However, electrodialysis involves anion exchange membranes (for acid separation) and is subject to limitations by fouling effects. It has been reported that a new technique, reverse electro-enhanced dialysis (REED) could substantially reduce the fouling effect [11, 12]. REED has so far been applied to lactic acid extraction [13] and recombinant protein production [14].

Despite the fact that the necessity for a bio-based economy has been in the forefront the last decade, studies on butyric acid production from second generation biomasses are scarce. Corn fiber hydrolysate [15], cane molasses [16] and Jerusalem artichoke [17] have so far been investigated as feedstocks for butyric acid production by immobilized *C. tyrobutyricum* in batch/fed-batch processes. Also, in a recent study by Liu et al. [18], hydrolysates of wheat straw, corn fiber, corn stover, rice hull and switch grass have been investigated as feedstock for butyric acid production by *C. tyrobutyricum* in batch tests. In that study, the glucose concentration in all hydrolysates was less than 40 g L^−1^, and experiments were running with diluted feedstocks so that the final butyric acid concentration was not exceeding 8 g L^−1^. The authors concluded that more research was needed to develop fed-batch/continuous fermentation processes with product removal to increase the titer of butyric acid.

The present study focuses on butyric acid fermentation of pre-treated (by wet explosion) and enzymatically hydrolysed wheat straw (PHWS). Application of continuous fermentation mode and in situ acid removal by REED was investigated in order to enhance the sugar consumption rates and butyric acid productivity.

## Materials and Methods

### Microorganism

*Clostridium tyrobutyricum,* strain DSMZ 2637 was obtained from Deutsche Sammlung von Microorganismen und Zellkulturen (DSMZ) and it was adapted to PHWS through subsequent transfers to increasing concentrations of PHWS as described by Baroi et al. [19]. The adapted stain was stored at −80 °C in 10 % glycerol and used throughout this study.

### Growth Medium and Biomass

Pretreated and hydrolyzed wheat straw (PHWS) was provided by the partner company Biogasol^®^, Denmark. The hydrolysate was produced during biorefinery processing of wheat straw from wheat grown and harvested in Denmark in 2011. The initial chemical composition of the wheat straw was 32.5 wt% cellulose, 26.4 wt% hemicellulose, 34.5 wt% lignin, 3.5 wt% ash and 3.0 wt% other compounds based on compositional analysis [20]. The wheat straw used in this work was processed by (1) pretreatment, (2) enzymatic hydrolysis and (3) solid/liquid separation. The pretreatment process was carried out at 163 °C for 15 min in a BioGasol Carbofrac™ 5D at the premises of BioGasol (Ballerup, Denmark) with sulphuric acid (1.4 wt% in the fluidization) as catalyst. The pretreatment was used to release the hemicellulose into the liquid fraction and to keep the lignin in the solid fraction [21]. To continue with enzymatic hydrolysis, the pretreated wheat straw was cooled down and water added to give a total solid content of 20 wt% and pH was adjusted to pH 5.0 with sodium hydroxide. Enzymatic hydrolysis was carried out as described in Öhrman et al. [22]. Prior to the fermentation experiments, PHWS was passed through an 8 μm (50 μm for the pilot experiment) pore-size filter for removing any remaining solids.

The growth medium used for the dilution of PHWS was as described by O’brien and Morris [23] and consisted of (per litre): 0.2 g MgSO_4_ 7H_2_O; 0.0076 g MnSO_4_ H_2_O; 0.01 g FeSO_4_ 7H_2_O; 4 g casein hydrolysate; 1 mg PABA; 2 µg biotin; 1 mg thiamine HCl. The growth medium was prepared under constant nitrogen gas flushing.

As the inorganic nitrogen (NH_3_-N) and phosphorus (PO_4_^−3^-P) concentrations in PHWS were insufficient to support the microorganism during fermentation of 100 % PHWS, nitrogen and phosphorus were added as urea and K_2_HPO_4_ based on the ratio COD/N/P = 400/7/1 [24] whenever experiments with 100 % PHWS were performed.

### Reverse Electro Enhanced Dialysis: REED Technology

A detailed description of REED can be found in Garde [13], Rype and Jonsson [11] and Prado-Rubio et al. [12]. The REED system was provided by Jurag Separation A/S.

Acids separated by REED were collected in Na-salt form. Dialysate and electrolyte were NaOH solutions. Fermentation broth and dialysate were recirculated at a speed of 400 and 200 ml min^−1^ respectively. Disinfection of the REED system and pipes was performed by circulating 400 ppm peracetic acid solution for 60–90 min followed by circulation of 10 L of sterile de-ionized water for washing-out the disinfectant from the system. pH, maximum current and voltage were set for 7, 5 A and 10 volt respectively. The REED extraction efficiency was calculated as following (Eq. 1):

1 $$ {\text{REED}}\,{\text{Extraction}}\,{\text{Efficiency}},\,\% = \frac{{{\text{Butyric}}\,{\text{acid}}\,{\text{extracted}}}}{{{\text{Total}}\,{\text{butyric}}\,{\text{acid}}}} \times 100 $$

### Batch and Continuous Fermentations

Batch and continuous bench-scale experiments were performed in a 1.5-L active volume (3-L total volume) Applikon^®^ autoclavable glass reactor equipped with a controller for pH, temperature and agitation. Sterilization of medium was performed by autoclaving at 121 ^°^C for 30 min whereas gas sterilization was carried out using a 0.2 µm Midistar^®^ 2000 PTFE gas filter. The fermentation was carried out at 37 ^°^C and agitation at 150 rpm and pH was maintained at 7 with 4 M KOH. Prior to fermentation, the controller was calibrated for pH and temperature. To remove oxygen, nitrogen gas was sparged into the reactor through a sterile gas filter. Outlet gas from the reactor was passed through a condenser connected on top of the reactor and a sterile gas filter and measured by a gas meter. The total pressure in the reactor was assumed to be 1 atm. In continuous operating mode, the influent and effluent flow was controlled by peristaltic pumps connected to the Applikon^®^ controller and the active volume was controlled by a level sensor.

An Applikon^®^ 20 L, stainless steel, pilot plant bioreactor system was used for the pilot scale experiment. The basic fermentation conditions in the pilot bioreactor were the same as for the bench scale experiments (37 ^°^C, agitation at 150 rpm and pH 7).

**Fig. 1:**
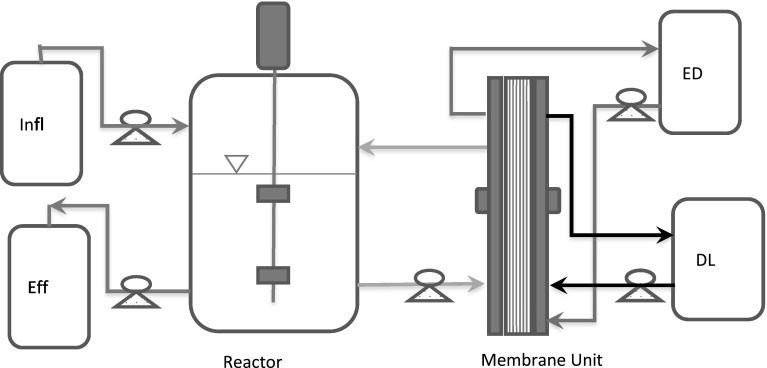
Schematic diagram of the experimental setup of continuous fermentation and in situ separation by REED. *Infl* inflow, *Eff* effluent, *ED* electrolyte, *DL* dialysate. The *green line* represents the fermentation broth circulation from reactor to membrane and back to reactor, and the *black* and *brown line* shows the dialysate and electrolyte circulation, respectively

The fermentors (bench-scale and pilot-scale) were connected to the REED membrane unit as shown in Fig. 1, for performing experiments with in situ acids removal.

### Stoichiometric Calculations

Stoichiometric calculations were based on product yields and calculation of the glucose and xylose electron equivalents partitioned between energy production (catabolism of glucose and xylose to various products) and biomass synthesis [26]. Assuming glucose and xylose as the sole electron donors in our experiments and calculating the fraction of electron equivalents found in each of the products the theoretical energy reaction was constructed. The organic half-reactions used for the substrates (glucose and xylose) and products (hydrogen, butyric and acetic acids) are as following (Eqs. 2–6):

2 $$ \frac{1}{24}C_{6} H_{12} O_{6} + \frac{1}{4}H_{2} O \to \frac{1}{4}CO_{2} + H^{ + } + e^{ - } $$

3 $$ \frac{1}{20}C_{5} H_{10} O_{5} + \frac{1}{4}H_{2} O \to \frac{1}{4}CO_{2} + H^{ + } + e^{ - } $$

4 $$ H^{ + } + e^{ - } \to \frac{1}{2}H_{2} $$

5 $$ \frac{4}{20}CO_{2} + H^{ + } + e^{ - } \to \frac{1}{20}CH_{3} CH_{2} CH_{2} COOH + \frac{6}{20}H_{2} O $$

6 $$ \frac{2}{8}CO_{2} + H^{ + } + e^{ - } \to \frac{1}{8}CH_{3} COOH + \frac{2}{8}H_{2} O $$

The fraction of the electron donors’ electron equivalents used for energy production (f_e_) was calculated from the difference between the product yields predicted by the theoretical energy reaction and the actual measured yields as reported in Antonopoulou et al. [27]. The fraction of the electron donors’ electron equivalents used for cell synthesis (f_s_) was then calculated using the Eq. 7:

7 $$ f_{s} + f_{e} = 1 $$

Subsequently, the microbial cell synthesis reaction was constructed for the experiment with 100 % PHWS using the cell formation half-reaction (8), NH_3_ as nitrogen source (urea was added as nitrogen source) and C_5_H_7_O_2_N as empirical formula for microbial cells. Glucose and xylose were again the sole carbon and energy sources.

8 $$ \frac{1}{5}CO_{2} + \frac{1}{20}HCO_{3}^{ - } + \frac{1}{20}NH_{4}^{ + } + H^{ + } + e^{ - } \to \frac{1}{20}C_{5} H_{7} O_{2} N + \frac{9}{20}H_{2} O $$

The overall stoichiometric reaction was finally constructed as the sum of the energy and cell synthesis reactions multiplied by f_e_ and f_s_, respectively, as described in Rittmann and McCarty [26].

### Analytical Methods

Sugars, 5-HMF and 2-furfural were quantified with HPLC-RI equipped with an Aminex HPX-87H column (BioRad) at 60 °C. A solution of 4 mmol L^−1^ H_2_SO_4_ was used as eluent at a flow rate of 0.6 ml min^−1^. Approximately 1 mL of liquid sample was acidified with 30 µL of 2 M H_2_SO_4_ to pH < 1.5 and centrifuged at 10,000 rpm for 10 min. The supernatant was filtrated through a 0.45 µm pore size filter. Acetic and butyric acids were quantified by gas chromatography (Perkin Elmer 400) using flame ionization detector and a SUPELCO polar fused silica 0.53 ID column. The temperature of the injection port was 240 °C, column temperature was set to 105 °C for 3 min and then increased to 230 °C in two steps, first with ramp of 8 °C per minutes to 130 °C and then final ramp of 45 °C per minutes to 230 °C for 3 min. Detector temperature was set to temperature at 240 °C. The carrier gas was nitrogen at a flow rate of 13 ml min^−1^. Prior to analysis, samples were acidified with 17 % H_3_PO_4_ to pH 2–3 and centrifuged at 10,000 rpm for 10 min. The supernatant was collected and filtered through a 0.45 µm pore size filter. Phosphorus and nitrogen were measured according to standard methods [25]. Total and volatile (TS, VS) solids and total suspended and volatile suspended (TSS, VSS) solids were analysed according to standard methods [25]. Gas composition in hydrogen was measured by a gas chromatograph (SRI 310C) equipped with a thermal conductivity detector and a packed column (Porapak-Q, length 6 ft and inner diameter 2.1 mm). The temperature for injector, column and detector was set to 80 °C.

## Results and Discussion

### Composition of PHWS

The composition of the PHWS used in the present study is shown in Table 1. PHWS consisted mainly of glucose and xylose (90 g L^−1^ in total) and it also contained small amounts of arabinose and cellulose. Acetic acid and 2-furfural, generated mainly from the pretreatment applied, were also present in low concentrations, while 5-hydroxy methyl furfural (5-HMF) was not detected.

**Table 1:** Composition of PHWS

Component	PHWS
Glucose (g L^−1^)	55.07 ± 0.10
Xylose (g L^−1^)	34.80 ± 0.16
Arabinose (g L^−1^)	3.92 ± 0.05
Cellobiose (g L^−1^)	1.40 ± 0.21
Acetate (g L^−1^)	4.52 ± 0.23
5-HMF (g L^−1^)	ND
2-Furfural (g L^−1^)	0.20 ± 0.09
Inorganic P (g L^−1^)	0.108 ± 003
Inorganic N (g L^−1^)	0.051 ± 0.001
TS (%)	12.94 ± 0.03
VS (%)	11.88 ± 0.03
pH	4.9 ± 0.2

*ND* not detectable

### PHWS Fermentations with *C. tyrobutyricum* Without In-Situ Acids Removal

As discussed by Baroi et al. [19], batch fermentations of PHWS by an adapted strain of *C. tyrobutyricum* exhibited very low glucose consumption rates and even lower, almost negligible xylose consumption rates although butyric acid yield and selectivity were quite satisfactory. Specifically, the butyric acid yield was 0.46 and 0.39 g g^−1^ sugars with a selectivity of 0.90 and 0.92 g g^−1^ acids, during batch experiments with 60 and 70 % PHWS, respectively. On the other hand, average glucose consumption rates were 212 and 71 mg L^−1^ h^−1^ at 60 and 70 % PHWS, respectively, while xylose consumption rates were even lower, 19 and 15 mg L^−1^ h^−1^, respectively [19]. Low sugar consumption rates resulted in low butyric acid productivities (average of 0.11 and 0.03 g L^−1^ h^−1^ during batch fermentation of 60 and 70 % PHWS) despite the high yield and selectivity achieved. Therefore, continuous operating mode was investigated as a way to increase sugar consumption rates and consequently butyric acid productivity.

Five continuous fermentation experiments were performed in total, with increasing concentration of PHWS (60–80 %) at HRTs within the range of 1–2 days, as following:

1st experiment: 60 % PHWS as influent at 1 day HRT

2nd experiment: 60 % PHWS as influent at 2 days HRT

3rd experiment: 70 % PHWS as influent at 2 days HRT

4th experiment: 80 % PHWS as influent at 2 days HRT

5th experiment: 80 % PHWS as influent at 1.5 days HRT

An HRT of 1 day was applied as starting point with 60 % PHWS in the influent. As there was a high residual xylose concentration in the fermentor, an HRT of 2 days was applied for subsequent experiments with 60, 70 and 80 % PHWS in the influent. 80 % PHWS resulted in significantly reduced xylose consumption rate and therefore higher concentrations of PHWS were not tested; however a lower HRT of 1.5 days was also applied with 80 % PHWS.

**Fig. 2:**
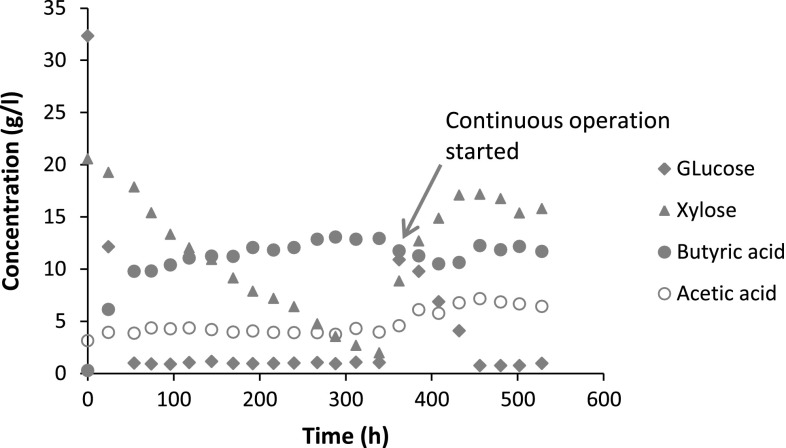
Sugars and acids concentration profiles during fermentation of 60 % PHWS by *C. tyrobutyricum*

Fermentation experiments were started-up in batch mode. The fermentor was initially operated in continuous mode once xylose was almost totally consumed and it was allowed to reach steady state. Glucose, xylose, butyric and acetic acid concentration and hydrogen produced were measured at each steady state. In Fig. 2, one can see the substrate and product concentration profiles (including the batch activation phase) until steady state was reached for the experiment with 60 % PHWS. Similar curves were obtained from all the above-mentioned experiments. Glucose and xylose concentrations and consumption rates, hydrogen, butyric and acetic acid production rates and yields obtained at steady states are shown in Table 2.

**Table 2:** Characteristics of the steady states during continuous fermentations with increasing concentration of PHWS

	60 % PHWS 1 day HRT	60 % PHWS 2 days HRT	70 % PHWS 2 days HRT	80 % PHWS 2 days HRT	80 % PHWS 1.5 days HRT
Glucose concentration (g L^−1^)	< 0.45	< 0.45	< 0.45	< 0.45	< 0.45
Xylose concentration (g L^−1^)	15.96	7.09	11.26	15.83	17.67
Butyric acid concentration (g L^−1^)	11.88	14.88	18.53	18.07	15.48
Acetic acid concentration (g L^−1^)	6.622	5.63	5.95	6.83	6.92
Glucose consumption rate^a^ (g L^−1^ h^−1^)	1.278	0.686	0.831	0.989	1.150
Xylose consumption rate (g L^−1^ h^−1^)	0.059	0.278	0.241	0.199	0.153
Acetic acid production rate^b^ (g L^−1^ h^−1^)	0.186	0.079	0.072	0.094	0.121
Acetic acid yield^b^ (g g^−1^)	0.139	0.082	0.068	0.079	0.094
Butyric acid production rate (g L^−1^ h^−1^)	0.552	0.399	0.454	0.477	0.507
Butyric acid yield (g g^−1^ sugars)	0.413	0.414	0.424	0.401	0.390
Butyric acid selectivity^c^ (g g^−1^ acids)	0.75	0.84	0.86	0.83	0.81
Hydrogen production rate (L L^−1^ h^−1^)	0.142	0.201	0.223	0.292	0.305

^a^Totally consumed (kinetically non-limited), highest possible rate achieved

^b^Yield and production rate of acetic acid was calculated based on the amount metabolized by the strain

^c^Butyric acid selectivity was calculated as the ratio of butyric acid yield to the sum of butyric acid and acetic acid yields

For all PHWS concentrations and HRT tested, glucose was totally consumed (<0.45 g L^−1^). Consumption of xylose was kinetically limited at the HRTs tested as the residual xylose concentration at steady state implied, however, xylose consumption rates were significantly higher than those obtained during the batch experiment (0.059–0.278 g L^−1^ h^−1^ compared to 0.019 g L^−1^ h^−1^ during batch fermentation of 60 % PHWS). Also, glucose consumption rates were substantially higher when continuous mode was applied (0.686–1.278 g L^−1^ h^−1^ compared to 0.212 g L^−1^ h^−1^ during batch fermentation of 60 % PHWS). It is obvious that the positive effect of continuous processing was greater in the case of xylose consumption rate. One can also notice that the concentration of glucose in the fermentor was very low during continuous processing, which could imply that increased glucose concentration as found during batch processing negatively affect the rate of xylose consumption. A similar phenomenon has been reported for *Thermoanaerobacterium thermosaccharolyticum* W16 grown on a glucose and xylose mixture [28]. The authors showed that the presence of glucose affected the consumption of xylose negatively compared to experiments where the microbial strain was grown solely on xylose.

The highest butyric acid productivity, 0.552 g L^−1^ h^−1^ was obtained during the 60 % PHWS fermentation at 1 day HRT and it was fivefold higher than the productivity obtained in batch mode. Butyric acid yields were maintained at the high levels achieved during batch fermentations (0.39–0.42 g g^−1^ sugars). Butyric acid selectivity was albeit lower, (0.75–0.86 g g^−1^ acids). Also, the lowest butyric acid selectivity was obtained at the lowest HRT tested (1 day), which is in agreement with the results of Michel-Savin et al. [5], who performed continuous experiments with *C. tyrobutyricum* grown on a glucose-based synthetic medium.

As it can be seen in Table 2, the concentration of butyric acid at steady state was in the range of 12 to 19 g L^−1^ while acetic acid concentration was 6 g L^−1^. Consequently, potassium ions (added in the form of potassium hydroxide for neutralization of the fermentation broth) concentration was 9 to 13 g L^−1^. Butyric acid has been reported to block growth of *C. tyrobutyricum* at 40 g L^−1^, while even 10 g L^−1^ cause significant inhibition [29]. It is known that in general, moderate concentrations (200–400 mg L^−1^) of potassium cations stimulate microbial growth while excessive amounts are inhibitory [30]. In order to minimize any inhibition caused by either butyric acid or potassium ion concentration or both, continuous fermentations with in situ acid removal by REED for achieving even higher butyric acid productivities were carried out.

### Continuous PHWS Fermentations with *C. tyrobutyricum* and In Situ Acid Removal by REED

**Fig. 3:**
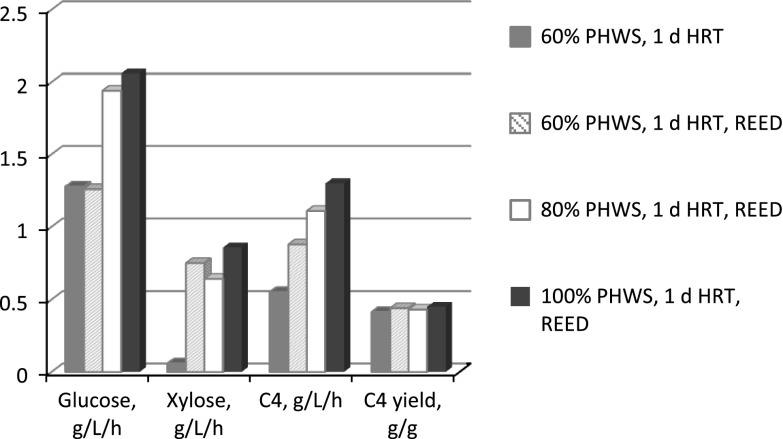
Glucose and xylose consumption rates and butyric acid (C4) production rate and yield obtained in the continuous experiments of PHWS with and without in situ acids removal by REED

The REED technology applied for in situ removal of acids is a membrane separation process and it combines elements from electrodialysis reversal and Donnan dialysis operations. In the present study, the REED membrane stack was equipped with anion-exchange (AX-REED) membranes for transport of anions. The AX-REED system continuously removes acid ions from the fermentation broth by replacing them with hydroxide ions. This ion exchange provides also pH regulation of the fermenter. Thus, the usual practice of regulating the pH by adding a strong base (NaOH or KOH) is avoided and inhibition from cations, especially when high concentrations of acids are produced, is prevented. Continuous fermentations with *C. tyrobutyricum* at increasing concentrations of PHWS (60, 70, 80 and 100 %) were conducted at a dilution rate of 0.0417 h^−1^ (HRT of 1 day). The fermentor was connected in-line with the REED system, allowing for in situ removal of produced acids. Glucose and xylose consumption rates, hydrogen, butyric and acetic acid production rates and yields along with REED extraction efficiencies are shown in Table 3. In Fig. 3, direct comparison of glucose and xylose consumption rates and butyric acid productivity and yield during PHWS fermentations with and without REED is illustrated.

**Table 3:** Characteristics of the steady states during continuous fermentations with increasing concentration of PHWS at 1 day HRT and in situ acids removal by REED

	60 % PHWS 1 day HRT	70 % PHWS 1 day HRT	80 % PHWS 1 day HRT	100 % PHWS 1 day HRT
Glucose concentration (g L^−1^)	< 0.45	< 0.45	< 0.45	< 0.45
Xylose concentration (g L^−1^)	1.99	1.33	5.83	1.07
Butyric acid concentration (g L^−1^)	3.30	3.51	5.83	6.97
Acetic acid concentration (g L^−1^)	0.72	0.81	0.95	0.64
Glucose consumption rate^a^ (g L^−1^ h^−1^)	1.26	1.37	1.94	2.06
Xylose consumption rate (g L^−1^ h^−1^)	0.75	0.80	0.64	0.86
Acetic acid production rate^b^ (g L^−1^ h^−1^)	0.15	0.17	0.13	0.16
Acetic acid yield^b^ (g g^−1^)	0.07	0.08	0.05	0.06
Butyric acid production rate (g L^−1^ h^−1^)	0.88	0.88	1.11	1.30
Butyric acid yield (g g^−1^)	0.44	0.41	0.43	0.45
Butyric acid selectivity^c^ (g g^−1^ acids)	0.86	0.84	0.90	0.88
Hydrogen production rate (L L^−1^ h^−1^)	0.41	0.47	0.52	0.58
REED extraction efficiency (%)	90.12	85.18	77.74	92.93

^a^Highest possible rate achieved (kinetically non-limited)

^b^Yield and production rate of acetic acid was calculated based on the amount metabolized by the strain

^c^Butyric acid selectivity was calculated as the ratio of butyric acid yield to the sum of butyric acid and acetic acid yields

The xylose consumption rate was enhanced by a factor of 12.5 when REED was applied during continuous fermentation of 60 % PHWS at 1 day HRT compared to the continuous process without in situ acids removal (Table 2, 1st experiment). At higher concentrations of PHWS, continuous processing with in situ acid removal was also more efficient than the continuous processing without REED, even at lower HRTs. The glucose consumption rate was also positively affected by REED at higher concentration of PHWS, although to a lower extent than that of xylose (Fig. 3). Butyric acid productivity was also significantly enhanced (Fig. 3). The highest butyric acid productivity (1.3 g L^−1^ h^−1^) was obtained when 100 % PHWS was fermented continuously with in situ acid removal (Table 3). In-situ acid removal by REED did not affect the butyric acid yield while butyric acid selectivity (0.84–0.90 g g^−1^ acids) was higher compared to the continuous processing without REED (0.75–0.86 g g^−1^ acids). Overall, continuous processing combined with in situ acid removal allowed for simultaneous consumption of both sugars at an HRT of 1 day resulting in almost 100 % sugars utilization. This is considered of great importance as xylose is a significant fraction of the sugars content of lignocellulosic biomasses and therefore, complete utilization of xylose along with glucose is a major step towards a cost-effective biological production process.

Also, continuous processing combined with in situ acid removal resulted in the same high butyric acid yield and much higher butyric acid productivity than that achieved in batch fermentations discussed in the beginning of the “Results and Discussion” section of the present manuscript. Specifically, the combined continuous fermentation mode and the in situ acid removal by REED resulted in 6- and 39-fold higher glucose and xylose consumption rates of 60 % PHWS, respectively, compared to those obtained by batch processing (212 and 19 mg L^−1^ h^−1^ for glucose and xylose, respectively). At 70 % PHWS, the enhancement was even higher (19- and 53-fold) for glucose and xylose consumption rates, respectively (71 and 16 mg L^−1^ h^−1^ for glucose and xylose, respectively, obtained during batch processing). This resulted in a 29-fold increase in butyric acid productivity when continuous fermentation of 70 % PHWS combined with REED was applied compared to batch processing. Also, it is interesting that the fermentation of 100 % PHWS proceeded unhindered with urea and K_2_HPO_4_ added as the only supplements. The reduced extraction efficiency observed at the experiment with 80 % PHWS was due to the fact that the membrane had been damaged and it was restored when the membrane was cleaned before the experiment with 100 % PHWS was conducted. However, despite that the extraction efficiency of the membrane was somewhat reduced (77 %) compared to the rest of the experiments (85, 90 and 92 %), acid-removal was still satisfactory as the concentrations of butyric and acetic acids in the fermentor were low and comparable to the rest of the experiments. Therefore, calculated rates and yields obtained from the experiment with 80 % PHWS could still be used for comparison purposes.

**Fig. 4:**
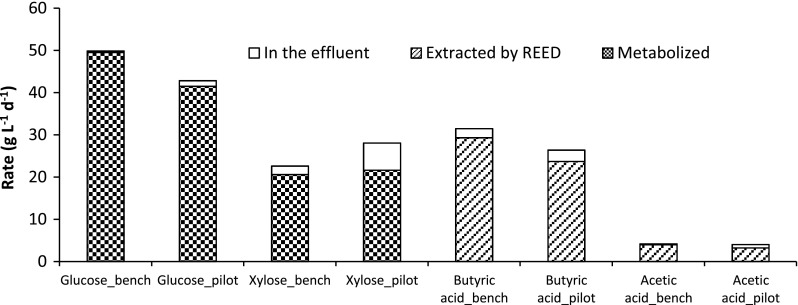
Rates of glucose and xylose metabolised and non-metabolised (in the effluent), and production and extraction rates of acetic and butyric acids at steady state during fermentation of 100 % PHWS in bench- and pilot-scale fermentors

In order to confirm the obtained results at a larger scale, continuous fermentation of 100 % PHWS with in situ acid removal was performed in the 20-L fermentor at 1.28 days HRT. Glucose and xylose consumption rates, hydrogen, butyric and acetic acids production rates and yields along with REED extraction efficiencies are shown in Table 4. In Fig. 4, comparisons of the bench-scale to the pilot-scale fermentation are made in terms of glucose and xylose consumption rates and butyric and acetic acid productivities. Extraction rates by REED are also shown in Fig. 4. The results of the bench- and pilot-scale were comparable; the lower glucose consumption and butyric acid production rates at the pilot-scale can be explained by the higher HRT applied.

**Table 4:** Characteristics of the steady states during continuous fermentations with 100 % (v/v) PHWS at 1.28 days HRT and in situ acids removal by REED in pilot scale

	100 % PHWS 1.28 days HRT
Glucose concentration (g L^−1^)	1.88
Xylose concentration (g L^−1^)	9.88
Butyric acid concentration (g L^−1^)	4.59
Acetic acid concentration (g L^−1^)	1.32
Glucose consumption rate (g L^−1^ h^−1^)	1.73
Xylose consumption rate (g L^−1^ h^−1^)	0.90
Acetic acid production rate^a^ (g L^−1^ h^−1^)	0.12
Acetic acid yield^a^ (g g^−1^)	0.05
Butyric acid production rate (g L^−1^ h^−1^)	1.10
Butyric acid yield (g g^−1^)	0.42
Butyric acid selectivity^b^ (g g^−1^ acids)	0.90
REED extraction efficiency (%)	89.66

^a^Yield and production rate of acetic acid was calculated based on the amount metabolized by the strain

^b^Butyric acid selectivity was calculated as the ratio of butyric acid yield to the sum of butyric acid and acetic acid yields

In conclusion, continuous fermentation coupled with in situ product removal by REED successfully addressed the challenges of low consumption rates of sugars especially that of xylose at batch mode and end-product inhibition. Therefore, it is a really promising direction for biological butyric acid production from wheat straw hydrolysate. From an industrial point of view, the developed process would be interesting in at least two perspectives: low feed cost and elimination of costly nutrients. Besides that, important future directions for fermentative butyric acid production would be investigating in depth the possibility of removing the gas phase for enhancing sugar consumption. Also, combining continuous operation, in situ product removal with cell recycle or attached microbial growth, as it has been shown [16, 17, 29] that microbial cells recycle and relevant reactor configurations positively influence to a great extent butyric acid productivity.

### Stoichiometric Analysis

Stoichiometric equations representing the energy reactions for the bench scale continuous experiments are shown in Table 5. One can observe that although REED allowed for higher xylose stoichiometric consumption and enhanced butyric acid production, it did not significantly influence the f_e_ and f_s_ values (for experiments without REED, the f_e_ and f_s_ were 0.911 ± 0.007 and 0.089 ± 0.007, respectively, while for experiments with REED the f_e_ and f_s_ were 0.923 ± 0.021 and 0.077 ± 0.021, respectively). This implies that the fraction of electrons used for maintenance and cell synthesis (represented by f_s_) is the same whether REED is applied or not. From an energetic point of view, higher butyric acid and potassium ion concentrations are anticipated to increase the maintenance requirements of the cells and therefore decrease the microbial cell net yields since the f_s_ value remains actually the same. Consequently, removal of acids by REED is anticipated to result in a larger number of microbial cells in the fermentor and higher substrate consumption rates, which fully complies with the measured tendency. Based on the stoichiometric calculations described in the “Materials and Methods” section, the continuous biological butyric acid production from 100 % PHWS followed the overall stoichiometric equation:

9 $$ \begin{aligned} C_{6} H_{12} O_{6} + 0.501C_{5} H_{10} O_{5} + 0.110HCO_{3}^{ - } + 0.110NH_{4}^{ + } \to 0.110C_{5} H_{7} O_{2} N + 2.074H_{2} + \hfill \\ + 1.291CH_{3} CH_{2} CH_{2} COOH + 0.233CH_{3} COOH + 0.692H_{2} O + 2.437CO_{2} \hfill \\ \end{aligned} $$

**Table 5:** Stoichiometric coefficients for the energy reactions applied for *C. tyrobutyricum growth* on increasing ratios of PHWS to synthetic growth medium at steady states with and without in situ acids removal by REED

	Reactants			Products					Electron fractions	
	C_6_H_12_O_6_	C_5_H_10_O_5_	H_2_O	H_2_	CH_3_CH_2_CH_2_COOH	CH_3_COOH	H_2_O	CO_2_	f_e_	f_s_
*Experiments without REED*										
60 % PHWS, 1d HRT	1	0.055	–	0.901	0.973	0.481	0.522	1.424	0.908	0.092
60 % PHWS, 2d HRT	1	0.485	–	2.358	1.300	0.377	0.121	2.478	0.915	0.085
70 % PHWS, 2d HRT	1	0.348	–	2.156	1.219	0.284	0.141	2.297	0.917	0.083
80 % PHWS, 2d HRT	1	0.241	0.110	2.378	1.079	0.312	–	2.268	0.914	0.086
80 % PHWS, 1.5d HRT	1	0.160	0.083	2.171	1.002	0.351	–	2.088	0.900	0.100
*Experiments with REED*										
60 % PHWS, 1d HRT	1	0.714		2.533	1.510	0.377	0.243	2.777	0.946	0.054
70 % PHWS, 1d HRT	1	0.701		2.800	1.456	0.412	0.056	2.856	0.903	0.097
80 % PHWS, 1d HRT	1	0.340		2.176	1.290	0.222	0.202	2.378	0.907	0.093
100 % PHWS, 1d HRT	1	0.501		2.217	1.380	0.249	0.271	2.488	0.936	0.064

## Conclusions

A process has been developed for fermentative butyric acid production by *C. tyrobutyricum* on PHWS based on continuous operation mode and in situ acids extraction by REED. Different dilutions of PHWS in a synthetic medium (60–100 % v/v) were tested and compared in batch and continuous operation mode with and without REED. It was found that continuous fermentation of 60 % PHWS resulted in six and three times higher glucose and xylose consumption rates, respectively, compared to batch fermentation (1.270 and 0.060 g L^−1^ h^−1^ for glucose and xylose in continuous mode compared to 0.212 and 0.019 g L^−1^ h^−1^ for glucose and xylose in batch mode). The application of REED enhanced the sugars consumption rates and butyric acid productivity even further. Specifically, the combined continuous fermentation mode and in situ acid removal by REED resulted in 6- and 39-fold higher glucose and xylose consumption rates of 60 % PHWS (1.260 and 0.750 g L^−1^ h^−1^), respectively, compared to those obtained by batch processing. At 70 % PHWS, the enhancement was even higher (19- and 53-fold for glucose and xylose consumption rate, respectively).

Fermentation of 100 % PHWS continued unhindered with urea and K_2_HPO_4_ added as sole N- and P-source. Butyric acid production rate, yield and selectivity were 1.30 g L^−1^ h^−1^, 0.45 g g^−1^ sugars and 0.88 g g^−1^ acids, respectively. Accompanying products were acetic acid and hydrogen at 0.16 g L^−1^ h^−1^ and 0.58 L L^−1^ h^−1^, respectively. In order to confirm the obtained results at a larger scale, continuous fermentation of 100 % PHWS with in situ acid removal was also performed in a 20 L pilot plant bioreactor system. The results were comparable to those obtained from the bench-scale fermentors.
